# Supplementary material for: A Critical View of the Application of the APEX Software (Aqueous Photochemistry of Environmentally-Occurring Xenobiotics) to Predict Photoreaction Kinetics in Surface Freshwaters
Source: Molecules. 2019 Dec 18;25(1):9. doi: 10.3390/molecules25010009 (PMC7017383; doi:10.3390/molecules25010009)
Supplement: Supplementary file 1 [file molecules-25-00009-s001.zip › 3.2.4_gcc-4.4.0.zip!/3.2.4_gcc-4.4.0/3.2.4_gcc-4.4.0/doc/octave/html/interpreter/Using-Sparse-Matrices-in-Oct_002dFiles.html]

Using Sparse Matrices in Oct-Files - Untitled


Previous: Creating Sparse Matrices in Oct-Files,
Up: Sparse Matrices in Oct-Files


---

##### A.1.6.3 Using Sparse Matrices in Oct-Files

Most of the same operators and functions on sparse matrices that are
available from the Octave are equally available with oct-files.
The basic means of extracting a sparse matrix from an `octave_value`
and returning them as an `octave_value`, can be seen in the
following example

```
     octave_value_list retval;
     
     SparseMatrix sm = args(0).sparse_matrix_value ();
     SparseComplexMatrix scm =
         args(1).sparse_complex_matrix_value ();
     SparseBoolMatrix sbm = args(2).sparse_bool_matrix_value ();
     ...
     retval(2) = sbm;
     retval(1) = scm;
     retval(0) = sm;
```

The conversion to an octave-value is handled by the sparse
`octave_value` constructors, and so no special care is needed.
